# Verbesserungswürdige Versorgungslage bei seltenen endokrinologischen Erkrankungen in Deutschland am Beispiel des endogenen Cushing-Syndroms

**DOI:** 10.1055/a-2776-2466

**Published:** 2026-02-23

**Authors:** Linus Haberbosch, Martin Fassnacht, Martin Reincke, Anna Kabilka, Timo Effenberger, Thomas Hupp, Jan Tuckermann, Stephan Petersenn, Ilonka Kreitschmann-Andermahr

**Affiliations:** 1 Klinik für Endokrinologie, Diabetologie und Stoffwechselmedizin, Charité – Universitätsmedizin Berlin, BerlinGermany; 2 Medizinische Klinik und Poliklinik I, Lehrstuhl für Endokrinologie und Diabetologie, Universitätsklinikum Würzburg, WürzburgGermany; 3 Medizinische Klinik, LMU Klinikum, Ludwig-Maximilians-Universität, MünchenGermany; 4 SmartStep Data Institute GmbH, HamburgGermany; 5 IQVIA Commercial GmbH and Co OHG, FrankfurtGermany; 6 Institut für Molekulare Endokrinologie und Physiologie, Universität Ulm, Ulm Germany/ German Center for Child and Adolescent Health (DZKJ) partner site Ulm, UlmGermany; 7 ENDOC Praxis für Endokrinologie und Andrologie, HamburgGermany; 8 Klinik für Neurochirurgie und Wirbelsäulenchirurgie, Universitätsklinikum Essen, EssenGermany

**Keywords:** Endogenes Cushing's Syndrome, Seltene Erkrankung, Cortisol, ACTH, Sekundärdatenanalyse, Endogenous Cushing Syndrome, Rare disease, Cortisol, ACTH, Secondary data analysis

## Abstract

Die Versorgung von Patienten mit Seltenen Erkrankungen in Deutschland stellt eine Herausforderung dar; das endogene Cushing-Syndrom verdeutlicht diese Problematik. Eine Analyse der Versorgungssituation soll Verbesserungspotenziale aufzeigen. Eine systematische Literaturrecherche zur Versorgungssituation wurde mit Verordnungsdaten aus Deutschland und Frankreich sowie Ärztestatistiken aus europäischen Ländern verglichen. In Deutschland besteht eine geringere Versorgungsdichte mit Endokrinologen, eine niedrigere Behandlungsprävalenz als in Frankreich und eine längere Zeit bis zur Diagnosestellung als in anderen Ländern mit vergleichbar gut entwickelten Gesundheitssystemen. Um eine bedarfsgerechte Versorgung von Seltenen Erkrankungen zu gewährleisten, sind Investitionen in die Aus- und Weiterbildung von Spezialisten, wie Fachärzte für Endokrinologie, und in neue, sektorenübergreifende Versorgungsformen notwendig.

## Abstract (en)

The care of patients with rare diseases in Germany poses a challenge; endogenous Cushing’s syndrome highlights this issue. An analysis of the care situation aims to identify areas for improvement. A systematic literature search on the care situation was compared with prescription data from Germany and France as well as physician statistics from various European countries. In Germany, there is a low density of endocrinologists, a lower treatment prevalence than in France and a longer time to diagnosis than in other countries with well-developed healthcare systems. In order to guarantee need-based care for rare diseases, investments in the training and further education of specialists, such as endocrinologists, and in new, cross-sectoral forms of care are necessary.

## Einleitung

Die medizinische Versorgung von Patienten mit seltenen Erkrankungen stellt Gesundheitssysteme vor besondere Herausforderungen 1 2 . Als selten gelten Erkrankungen, wenn sie nicht mehr als 5 von 10 000 Menschen betreffen 3 . In Deutschland leben etwa 4 Millionen Menschen mit einer der rund 7 000 bis 8 000 klassifizierten seltenen Krankheiten 4 . Die Diagnostik ist häufig langwierig 2 4 , geprägt von Arztwechseln und einem hohen Bedarf an zusätzlicher Patientenunterstützung 5 .

Eine sehr seltene, unbehandelt lebensbedrohliche, Erkrankung ist das endogene Cushing-Syndrom mit einer geschätzten Inzidenz von 0,2 pro 100 000 und einer Prävalenz von 4,0 Patienten pro 100 000 Einwohner 6 . Es entsteht durch eine chronisch erhöhte endogene Cortisolproduktion 7 . Mit 60–70% ist die häufigste Form der Morbus Cushing (M. Cushing), verursacht durch ein corticotropes Hypophysenadenom (ACTH-produzierendes PitNET gemäß WHO-Klassifikation), das autonom adrenocorticotropes Hormon (ACTH) und so eine übermäßige Cortisolbildung in den Nebennieren verursacht 8 9 . Daneben gibt es das ektope Cushing-Syndrom (z. B. ACTH-produzierende Tumoren in der Lunge) sowie ACTH-unabhängige Formen durch Nebennierentumoren. Die primäre Therapie ist die operative Entfernung des Tumors, mit Remissionsraten von ca. 80% 7 10 . Bei Nichtansprechen können Reoperation, Bestrahlung, medikamentöse Therapie oder die beidseitige Adrenalektomie folgen 11 .

Betroffene entwickeln ein typisches Erscheinungsbild mit stammbetonter Gewichtszunahme, dünnen Extremitäten, Fettablagerungen am Hals («Büffelnacken»), rötlichen Dehnungsstreifen, Blutergüssen und Hirsutismus. Weitere Symptome sind Bluthochdruck, Diabetes mellitus, Osteoporose, Immunsuppression, Stimmungsschwankungen, psychischen Erkrankungen und Gedächtnisstörungen. Darüber hinaus können lebenslimitierende Komplikationen auftreten, wie thrombotische bzw. thromboembolische Ereignisse, Addison-Krisen nach operativen Eingriffen, schwere Infektionen, hypertensive Entgleisungen sowie metabolische Krisen im Rahmen von Diabetes mellitus 7 12 13 . Da sich die Veränderungen schleichend entwickeln und unspezifisch sind 13 14 , erfolgt die Diagnose erst Jahre nach Krankheitsbeginn. Patienten suchen häufig zuerst den Hausarzt auf, der aufgrund der Seltenheit des Syndroms charakteristische Symptomkombinationen oft nicht erkennt, mit teils schwerwiegenden Folgen.

Patienten mit Cushing-Syndrom haben ein bis zu dreifach erhöhtes Sterberisiko 15 ; unbehandelt liegt die 5-Jahres-Überlebensrate bei nur 50% 16 . Zusätzlich leiden sie unter erheblichen körperlichen und psychischen Belastungen, was ihre Lebensqualität verringert 17 . Eine frühzeitige, angemessene Diagnostik und Therapie können die Mortalität senken sowie die Lebenserwartung und -qualität verbessern. Dafür sind eine präzise Differentialdiagnose und eine zeitnahe Überweisung vom Hausarzt an einen Endokrinologen essenziell. Angesichts der hohen Krankheitslast ist eine ausreichende Zahl spezialisierter Fachärzte entscheidend.

Ziel dieser Arbeit war eine Analyse der Versorgungssituation von Patienten mit Cushing-Syndrom in Deutschland. Dazu wurde eine systematische Literaturrecherche durchgeführt und mit Verordnungsdaten sowie Ärztestatistiken aus Deutschland, Frankreich und weiteren europäischen Ländern in Beziehung gesetzt.

## Methodik

### Systematische Literaturrecherche

Zur Kartierung der Versorgungslandschaft von Patienten mit Cushing-Syndrom in Deutschland wurde zunächst eine systematische Literaturrecherche bei MEDLINE durchgeführt (zuletzt aktualisiert am 10. April 2024). Die Suchstrategie umfasste Freitextbegriffe als auch kontrollierte Vokabeln (MeSH-Terms): („Pituitary ACTH Hypersecretion“[Mesh] OR „Cushing Syndrome“[Mesh] OR „Cushing Syndrome“[tiab] OR Hypercortisolism[tiab] OR „Cushing Disease“[tiab]) AND (german*OR deutsch*). Die Auswahl relevanter Publikationen erfolgte durch zwei unabhängige Reviewer (TE, AK). In die Auswertung wurden deutsch- oder englischsprachige Publikationen der letzten 10 Jahre eingeschlossen, die Daten zur Über-, Unter- oder Fehlversorgung von Patienten mit einem Cushing-Syndrom in Deutschland enthielten. Systematische Übersichtsarbeiten, Fallstudien und Abstracts wurden ausgeschlossen, jedoch wurden die Referenzen gesichtet, um die Vollständigkeit des Studienpools sicherzustellen ( Abb. 1 ).

**Abb. 1:**
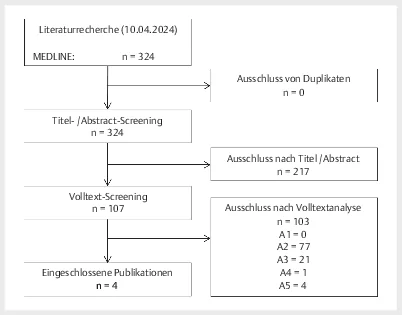
Flussdiagramm ein- und ausgeschlossener Publikationen. A1 – Population kein endogenes Cushing; A2 – Kein Bezug zu Über-, Unter-, Fehlversorgung; A3 – Publikationstyp (Fallstudie, Übersichtsarbeit, Abstract); A4 – Duplikat / Doppelte Population; A5 – keine Ergebnisse für deutsche Patienten.

### Behandlungsprävalenz basierend auf Verordnungsdaten

Die Schätzung der Behandlungsprävalenz in Deutschland und Frankreich erfolgte anhand longitudinaler Verordnungsdaten zu den am häufigsten eingesetzten Medikamenten bei Cushing-Syndrom. Dazu gehören Osilodrostat, Ketoconazol und Metyrapon. Pasireotid und Cabergolin wurden ausgeschlossen, da sie auch bei anderen Erkrankungen eingesetzt werden. Für beide Länder wurde der Zeitraum von Januar bis Dezember 2023 gewählt. Die Abdeckung der Verordnungsdaten als Datengrundlage variierte je nach Land.

Frankreich: Die Grundlage bildete ein Panel von ca. 10 000 Apotheken (insgesamt ca. 22 000), die Daten von 47 Millionen Patienten erfassen. Die Abgabe wird über einen eindeutigen, anonymen Schlüssel erfasst, unabhängig von der abgebenden Apotheke, um den Datenschutz zu gewährleisten.

Deutschland: Als Grundlage wurde die nationale Datenbank IQVIA Therapy Matrix Patient verwendet. Sie basiert auf Verordnungsdaten aus Offizinapotheken, die über Apothekenrechenzentren erfasst werden, und deckt etwa 80% der Verordnungen der Gesetzlichen Krankenversicherung ab. Die Projektion auf die bundesweite Verordnungsebene erfolgt durch den Abgleich mit dem bundesweiten Verordnungsvolumen aus IQVIA PharmaScope, das einen umfassenden Überblick über die Abgabe von Medikamenten über Offizin- und Versandapotheken, sowohl in der Gesetzlichen als auch in der Privaten Krankenversicherung ermöglicht.

### Analyse der endokrinologischen Versorgung im internationalen Vergleich

Statistische Daten zu Endokrinologen für die Jahre 2012 bis 2023 wurden aus verschiedenen nationalen Quellen bezogen. Für Deutschland wurde auf die Statistiken der Bundesärztekammer zurückgegriffen, wobei Endokrinologen aus den Fachgebieten Frauenheilkunde und Geburtshilfe sowie Kinder- und Jugendmedizin ausgeschlossen und nur solche aus dem Fachgebiet Innere Medizin berücksichtigt wurden 18 . Für die Schweiz wurden die Statistiken des Bundesverbandes der Schweizer Ärzte berücksichtigt (Bereich Endokrinologie und Diabetologie) 19 . Die Bevölkerungszahlen stammen aus entsprechenden nationalen Quellen 20 21 . Für Frankreich waren Daten zu den Ärzten im Fachgebiet Endokrinologie und Stoffwechsel in den Statistiken des französischen Gesundheitsministeriums verfügbar 22 . Zusätzlich wurden für Dezember 2024 die Anzahl aktiver Endokrinologen verschiedener europäischer Länder aus der IQVIA Reference Healthcare Professional Database entnommen. Mithilfe von Bevölkerungszahlen der Eurostat Statistiken bzw. der United Nations Population Division wurde die Anzahl der Endokrinologen pro Kopf berechnet 23 24 .

## Ergebnisse

### Systematische Literaturrecherche

Insgesamt wurden 324 Publikationen identifiziert, die anhand von Titel und Abstract unter Beachtung der prädefinierten Ein- und Ausschlusskriterien bewertet wurden. Es wurden 107 Volltexte untersucht und vier Studien eingeschlossen ( Abb. 1 ). Es ließen sich zwei zentrale Themenfelder identifizieren:

#### Zeit bis zur Diagnose und diagnostischer Prozess

Zwei Studien haben den zeitlichen Abstand zwischen dem Auftreten der ersten Symptome und der gesicherten Diagnose des Cushing-Syndroms oder M. Cushing untersucht. Rubinstein et al. 25 analysierten 5 367 Patienten weltweit, davon 172 in Deutschland, die zwischen 1986 und 2018 mit einem Cushing-Syndrom diagnostiziert wurden. Die Zeit bis zur Diagnosestellung betrug im Schnitt 4±4,25 Jahre und lag damit im internationalen Vergleich höher (2,83 Jahre). Kreitschmann-Andermahr et al. (2015) 26 untersuchten 176 Patienten, die zwischen 2005 und 2014 mit M. Cushing diagnostiziert wurden. Diese Studie berichtete, dass Patienten nach Symptombeginn durchschnittlich 0,9±2,0 Jahre warteten, bevor sie ärztlichen Rat suchten. Bis zur Diagnosestellung vergingen weitere 3,0±4,5 Jahre (Median: 1 Jahr). Patienten konsultierten bis zur Diagnose 4,6±3,8 verschiedene Ärzte, mit signifikanten Unterschieden zwischen städtischem und ländlichem Raum (4,2±4,0 vs. 5,1±3,5 Ärzte).

Eine Studie von Braun et al. 27 untersuchte, welche klinischen Leitsymptome Anlass zur Überweisung in eine universitäre endokrinologische Fachambulanz waren und inwiefern diese mit der internationalen Endocrine Society Practice Guideline aus dem Jahr 2008 13 übereinstimmten. Von 377 zugewiesenen Patienten wiesen 93 ein Cushing-Syndrom auf, bei 284 wurde die Erkrankung ausgeschlossen. Dabei erhöhten fünf Gründe für ein Screening die Wahrscheinlichkeit für das Vorliegen eines Cushing-Syndroms: Myopathie, metabolisches Syndrom, Osteoporose, Vorliegen eines Hypophysenadenoms und die Kombination mehrerer Cushing-Syndrom-spezifischer Symptome. Adipositas als Hauptbeschwerde verringerte hingegen die Wahrscheinlichkeit einer Cushing-Syndrom Diagnose. Die Autoren kamen zu dem Schluss, dass die Empfehlungen dieser Practice Guideline immer noch gültig sind, auch wenn das Vorgehen in der täglichen Praxis von diesen oft abweicht.

#### Krankheitslast und ungedeckte Unterstützungsbedürfnisse

Kreitschmann-Andermahr et al. (2018) 28 analysierten in einer Umfrage die Krankheitslast und Unterstützungsbedürfnisse von 71 Patienten mit M. Cushing in Deutschland. Sie zeigten, dass nur 44,4% der Befragten mit der medizinischen Versorgung zufrieden waren und ihre Ärzte im Umgang mit ihrer Erkrankung als kompetent und erfahren einstuften. Nur 12,7% hatten zu keinem Zeitpunkt ihrer Erkrankung den Wunsch nach weiterer Unterstützung. Etwa 45,1% der Patienten wünschten sich mehr Informationen über ihre Erkrankung, etwa in Form von Informationsblättern oder Broschüren.

### Behandlungsprävalenz

Die Analyse der Verordnungsdaten von 2023 ergab, dass sowohl die Patientenzahl (479 vs. 753 Patienten), als auch die Behandlungsprävalenz (5,73 vs. 11,01 pro Million) in Deutschland niedriger war als in Frankreich. Von den in Deutschland 479 Patienten, die mit Osilodrostat-, Ketoconazol- oder Metyrapon-Therapie behandelt wurden, waren 395 Patienten gesetzlich versichert.

### Zahl der Endokrinologen pro 100 000 Einwohner

Im Jahr 2012 betrug die Zahl der Endokrinologen in Deutschland 0,71 pro 100 000 Einwohner und stieg in den folgenden 10 Jahren bis 2023 leicht auf 0,89 an. Im Vergleich dazu lag die Anzahl in Frankreich im Jahr 2012 bei 2,67 pro 100 000 Einwohner und erhöhte sich bis 2023 auf 3,05. In der Schweiz stieg die Anzahl der Endokrinologen im gleichen Zeitraum von 2,16 auf 3,16 pro 100 000 Einwohner ( Abb. 2 ). Im internationalen Vergleich reiht sich Deutschland im Jahr 2024 mit 0,81 Endokrinologen pro 100 000 Einwohner hinter anderen nord- und südeuropäischen Staaten ein (1,43–6,27 pro 100 000) ( Tab. 1 ).

**Abb. 2:**
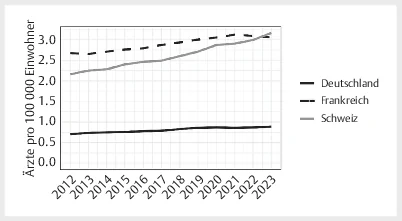
Vergleich Anzahl Endokrinologen gegenüber der Gesamtbevölkerung in Deutschland, Frankreich und der Schweiz.

**Table:** **Tab. 1** Anzahl an Endokrinologen pro 100 000 Einwohner im Jahr 2024 im internationalen Vergleich

Land	Endokrinologen pro 100 000 Einwohner
Dänemark	6,27
Italien	5,65
Spanien	4,92
Schweiz	4,80
Österreich	3,80
Frankreich	3,52
Norwegen	2,57
Polen	2,37
Finnland	1,43
*Deutschland*	*0,81*

## Diskussion

Die vorliegende Analyse basierend auf Literaturrecherche, Verordnungsdaten und Ärztestatistiken weist auf signifikante Versorgungsdefizite in der Diagnostik und Behandlung des endogenen Cushing-Syndroms in Deutschland hin.

Die Ergebnisse der systematischen Literaturrecherche zeigen, dass die Diagnose in Deutschland im Vergleich zu anderen Ländern deutlich später gestellt wird 25 26 . Mögliche Gründe hierfür sind, dass Patienten ihren Hausarzt oft mit unspezifischen Symptomen aufsuchen und, dass für die Bandbreite an Symptomen häufig unterschiedliche Fachärzte konsultiert werden. Dies in Kombination mit der Seltenheit des Cushings-Syndroms kann zu einer späten Diagnose beitragen 28 . Braun et al. 27 bestätigen, dass bei richtiger Einschätzung von Cushing-Symptomen sowie zeitnaher Überweisung zum Endokrinologen, eine relevante Anzahl von Patienten diagnostiziert werden kann.

Potenzielle Versorgungsdefizite werden auch durch die niedrigere medikamentöse Behandlungsprävalenz in Deutschland verdeutlicht. Allerdings sind länderspezifische Unterschiede, z. B. in Bezug auf die bilaterale Adrenalektomie, welche bei Therapieversagen sowie als Notfalltherapie bei schwersten Verläufen angewendet wird, zu berücksichtigen 29 : So gibt es in Frankreich eine Präferenz für eine medikamentöse Therapie 30 , während in Deutschland eine größere Offenheit gegenüber der operativen Therapie besteht 29 . Für Deutschland wird eine mittlere Zeit zur bilateralen Adrenalektomie von 7,5 Monaten 31 und für Frankreich von 6,1 Jahren bis zur Operation berichtet 32 .

Differenzen in den Verordnungen könnten weiterhin durch die unterschiedliche Versorgungssituation entstehen. Während der Zugang zur Grundversorgung ähnlich ist 33 34 , gab es 2023 in Frankreich 0,5 Neurochirurgen und in Deutschland 2,1 Neurochirurgen pro 100 000 Einwohner 33 34 . Demgegenüber stehen 0,81–0,89 Endrokrinologen pro 100 000 Einwohner in Deutschland und 3,05–3,52 in Frankreich. Die resultierende Versorgung von Patienten mit hypophysärem M. Cushing in neurochirurgischen Zentren könnte in Deutschland zu einer Bevorzugung der (Re)Operation gegenüber der medikamentösen Therapie führen. Der Unterschied in der endokrinologischen Versorgungsdichte kann teilweise durch stärkere Einbindung von Hausärzten in der deutschen Diabetesversorgung erklärt werden 35 .

Dennoch ist der eklatante Endokrinologenmangel in Deutschland bekannt 36 37 . Endokrinologen sind mit 69,9% die wichtigste ärztliche Anlaufstelle für die Diagnose und Behandlung des Cushing-Syndroms und nehmen durch ihre Expertise und enge Vernetzung eine entscheidende koordinative Rolle ein 26 . Studien belegen, dass der Mangel an Endokrinologen Versorgungsdefizite bei seltenen Erkrankungen wie dem Nebennierenrindenkarzinom verursacht, etwa durch verzögerte Diagnosen und unzureichende interdisziplinäre Betreuung 38 . Es ist davon auszugehen, dass auch bei weiteren seltenen endokrinen Erkrankungen eine suboptimale Versorgung besteht, die einer systematischen, vertieften Untersuchung bedarf. Gleichzeitig sollte das erhebliche Potenzial der Endokrinologen nicht unterschätzt werden, da sie neben seltenen Erkrankungen eine Vielzahl von Patienten mit häufigen Volkskrankheiten betreuen und somit eine zentrale Rolle in der integrierten Versorgung einnehmen. Die regionale Verteilung von Endokrinologen ist heterogen, was die Versorgungsdichte zusätzlich beeinflusst und Fragen zur Versorgungsqualität aufwirft, insbesondere in Regionen mit geringer fachärztlicher Infrastruktur 39 . Gerade im Kontext der zunehmenden Ambulantisierung der Medizin in Deutschland wird dies absehbar zu zunehmenden Versorgungsengpässen führen.

Neben seltenen Erkrankungen wie dem Cushing-Syndrom könnte eine eingeschränkte endokrinologische Versorgung auch häufiger auftretende, differenzialdiagnostisch oder pathogenetisch verwandte Krankheitsbilder betreffen. Dazu zählen beispielsweise Nebennieren- und Hypophysentumoren, Osteoporose, neuroendokrine Tumoren sowie metabolische Störungen wie Diabetes mellitus und Dyslipidämien. Sekundäre Diabetesformen, die etwa durch endokrine Erkrankungen ausgelöst werden können, aber auch Subtypen des Typ-2-Diabetes werden in einer primär hausärztlich organisierten Versorgung möglicherweise weniger häufig identifiziert und adäquat versorgt 40 .

Fachgesellschaften fordern daher den Erhalt und Ausbau von Lehrstühlen für Endokrinologie an den 37 staatlichen medizinischen Fakultäten in Deutschland, von denen im Jahr 2022 nur 7 Fakultäten eine eigenständige Klinik für Endokrinologie und Diabetologie enthielten 37 . Zudem ist die Zahl der Facharztweiterbildungsstellen in Deutschland limitiert. Dieser Mangel verschärft absehbar die schon bestehende geringe endokrinologische Versorgungsdichte.

Ein weiterer Faktor für die Versorgungsunterschiede könnten die unterschiedlichen Strategien im Umgang mit seltenen Erkrankungen sein. Während Frankreich bereits 2004 einen nationalen Plan für seltene Erkrankungen einführte, um Diagnose, Behandlung und Betreuung zu verbessern 41 , startete Deutschland erst 2013 einen entsprechenden nationalen Aktionsplan 35 41 . Dieser empfiehlt eine interdisziplinäre Versorgung (z. B. Fallkonferenzen oder in Zentren), damit Betroffene strukturiert und optimal versorgt werden können.

### Stärken und Schwächen

Eine Stärke dieser Arbeit ist die Repräsentativität der Verordnungsdaten, die detaillierte Vergleiche der Verschreibungsmuster in Deutschland und Frankreich ermöglicht und Einblicke in die Behandlungspraxis gibt. Eine weitere Stärke war die breit angelegte Suchstrategie ohne thematische Filter, die eine umfassende Analyse ermöglichte; zudem fokussiert auf die letzten 10 Jahre und darauf abzielend, den aktuellen Forschungsstand abzubilden.

Allerdings erlaubt die Analyse nur Vergleiche von medikamentös behandelten Patienten und nicht von absoluten Fallzahlen. Die unterschiedlichen Fachrichtungen und Berufsbezeichnungen sowie verschiedene Versorgungsstrukturen in den Ländern erschweren den Vergleich der Endokrinologenanzahlen. Die bereits erwähnte hohe Einbindung der Hausärzte in die Versorgung von Diabetes-Patienten in Deutschland könnte die Diskrepanzen relativieren. Die Fokussierung der Recherche auf MEDLINE birgt das Risiko, dass potenziell relevante Publikationen nicht berücksichtigt wurden, auch wenn dies als wenig wahrscheinlich einzustufen ist.

### Schlussfolgerungen

Zusammenfassend ist das endogene Cushing-Syndrom eine lebensbedrohliche, seltene Erkrankung, die in Deutschland deutlich unterdiagnostiziert ist, mit potentiell schwerwiegenden Folgen für die Lebenserwartung und Lebensqualität der Betroffenen. Eine stärkere Fokussierung auf diese Erkrankung ist dringend erforderlich, um das Mortalitätsrisiko sowie die gesundheitlichen und psychosozialen Belastungen durch verzögerte Diagnosestellung und Therapieeinleitung zu reduzieren.

Dazu braucht es einen multidimensionalen Ansatz: die strukturelle und personelle Stärkung der Endokrinologie im Medizinstudium, eine adäquate Aus- und Weiterbildung von Endokrinologen, die Schulung von Hausärzten in der Früherkennung seltener Krankheiten sowie den Einsatz von Registern und modernen Datenbanken mit geeigneten Filtern und Kommunikationstools.

Zudem sollte die Zahl der Endokrinologen mindestens das europäische Mindestniveau von 2–4 Fachärzten pro 100 000 Einwohner erreichen 42 . Dieses Ziel wird nur mit klaren gesundheitspolitischen Maßnahmen realisierbar sein. Eine sektorübergreifende Vernetzung, in der die Endokrinologie eine koordinative Rolle übernimmt, ist essenziell für eine optimale Versorgung.

## Fördermittel

Recordati Rare Diseases Germany GmbH

## References

[1] Nationales Aktionsbündnis für Menschen mit Seltenen Erkrankungen (NAMSE) Nationaler Aktionsplan für Menschen mit Seltenen Erkrankungen 2013 10.1007/s00103-017-2530-0 28337510

[2] Aichinger H Brkic N Schneider D Die gesundheitliche Situation von Menschen mit Seltenen Erkrankungen in DeutschlandEndbericht 2023

[3] Nguengang Wakap S Lambert D M Olry A Estimating cumulative point prevalence of rare diseases: analysis of the Orphanet database European Journal of Human Genetics 2020 28 165 173 10.1038/s41431-019-0508-0 PMC6974615 31527858

[4] Frank M Eidt-Koch D Aumann I Maßnahmen zur Verbesserung der gesundheitlichen Situation von Menschen mit seltenen Erkrankungen in Deutschland Bundesgesundheitsblatt – Gesundheitsforschung – Gesundheitsschutz 2014 57 1216 1223 10.1007/s00103-014-2040-2 25209683

[5] Depping M K Uhlenbusch N von Kodolitsch Y Supportive care needs of patients with rare chronic diseases: multi-method, cross-sectional study Orphanet journal of rare diseases 2021 16 1 10 10.1186/s13023-020-01660-w PMC7825171 33482869

[6] Orphanet. Prevalence and incidence of rare diseases: Bibliographic data2024

[7] Reincke M Fleseriu M Cushing Syndrome: A Review Jama. 2023 330 170 181 10.1001/jama.2023.11305 37432427

[8] Tritos N A Miller K K Diagnosis and management of pituitary adenomas: a review Jama. 2023 329 1386 1398 10.1001/jama.2023.5444 37097352

[9] Hakami O A Ahmed S Karavitaki N Epidemiology and mortality of Cushing's syndrome Best practice & research Clinical endocrinology & metabolism 2021 35 101521 10.1016/j.beem.2021.101521 33766428

[10] Petersenn S Beckers A Ferone D Therapy of endocrine disease: outcomes in patients with Cushing's disease undergoing transsphenoidal surgery: systematic review assessing criteria used to define remission and recurrence European Journal of Endocrinology 2015 172 R227 R39 10.1530/EJE-14-0883 25599709

[11] Fleseriu M Auchus R Bancos I Consensus on diagnosis and management of Cushing's disease: a guideline update The lancet Diabetes & endocrinology 2021 9 847 875 10.1016/S2213-8587(21)00235-7 PMC8743006 34687601

[12] Braun L T Vogel F Reincke M Long-term morbidity and mortality in patients with Cushing's syndrome Journal of neuroendocrinology 2022 34 e13113 10.1111/jne.13113 35312199

[13] Nieman L K Biller B M Findling J W The diagnosis of Cushing's syndrome: an endocrine society clinical practice guideline The Journal of Clinical Endocrinology & Metabolism 2008 93 1526 1540 10.1210/jc.2008-0125 PMC2386281 18334580

[14] Scaroni C Albiger N M Palmieri S Approach to patients with pseudo-Cushing’s states Endocrine connections 2020 9 R1 R13 10.1530/EC-19-0435 PMC6993268 31846432

[15] Limumpornpetch P Morgan A W Tiganescu A The Effect of Endogenous Cushing Syndrome on All-cause and Cause-specific Mortality. The Journal of clinical endocrinology and metabolism 2022 107 2377 88 10.1210/clinem/dgac265 PMC9282270 35486378

[16] Plotz C M Knowlton A I Ragan C The natural history of Cushing's syndrome The American journal of medicine 1952 13 597 614 10.1016/0002-9343(52)90027-2 12996538

[17] Kreitschmann-Andermahr I Morbus Cushing: Eine seltene endokrine Erkrankung mit schweren Folgen Forum Sanitas 2023 4 27 29

[18] Bundesärztekammer. Ärztestatistik 2023 [Available from:https://www.bundesaerztekammer.de/

[19] Bundesverband der Schweizer Ärzte. Berufstätige Ärzte nach Sektoren 2023 [Available from:https://fmh.ch/

[20] Statistisches Bundesamt Bevölkerung nach Altersgruppen (ab 2011) 2023 [Available from:https://www.destatis.de/DE/Themen/Gesellschaft-Umwelt/Bevoelkerung/Bevoelkerungsstand/Tabellen/liste-altersgruppen.html

[21] Schweizer Bundesamt für Statistik. Bevölkerungsdaten im Zeitvergleich, 1950 – 2022. 2022.

[22] social Lespese. [Demografie der Angehörigen von Gesundheitsberufen] 2023 [Available from:https://drees.solidarites-sante.gouv.fr/

[23] United Nations Population Division World Population Prospects 2024[Available from:https://www.un.org/development/desa/pd/world-population-prospects-2024

[24] Eurostat. Population on 1 Jan 2024 [Available from:https://ec.europa.eu/eurostat/web/main/

[25] Rubinstein G Osswald A Hoster E Time to Diagnosis in Cushing's Syndrome: A Meta-Analysis Based on 5367 PatientsThe Journal of clinical endocrinology and metabolism 2020 105 10.1210/clinem/dgz136 31665382

[26] Kreitschmann-Andermahr I Psaras T Tsiogka M From first symptoms to final diagnosis of Cushing's disease: experiences of 176 patients Eur J Endocrinol 2015 172 285 289 10.1530/EJE-14-0766 25501963

[27] Braun L T Vogel F Zopp S Whom Should We Screen for Cushing Syndrome? The Endocrine Society Practice Guideline Recommendations 2008 Revisited. The Journal of Clinical Endocrinology & Metabolism 2022 107 e3723 e3730 10.1210/clinem/dgac379 PMC9387700 35730067

[28] Kreitschmann-Andermahr I Siegel S Gammel C Support Needs of Patients with Cushing's Disease and Cushing's Syndrome: Results of a Survey Conducted in Germany and the USA International journal of endocrinology 2018 2018 9.014768E6 10.1155/2018/9014768 PMC6198616 30402098

[29] Reincke M Ritzel K Oßwald A A critical reappraisal of bilateral adrenalectomy for ACTH-dependent Cushing's syndrome* European Journal of Endocrinology 2015 173 M23 M32 10.1530/EJE-15-0265 25994948

[30] Dormoy A Haissaguerre M Vitellius G Efficacy and Safety of Osilodrostat in Paraneoplastic Cushing Syndrome: A Real-World Multicenter Study in France The Journal of Clinical Endocrinology & Metabolism 2022 108 1475 1487 10.1210/clinem/dgac691 PMC10188310 36470583

[31] Reibetanz J Kelm M Uttinger K L Differences in morbidity and mortality between unilateral adrenalectomy for adrenal Cushing's syndrome and bilateral adrenalectomy for therapy refractory extra-adrenal Cushing's syndrome Langenbeck's archives of surgery 2022 407 2481 2488 10.1007/s00423-022-02568-8 PMC9467939 35633419

[32] Sarkis P Rabilloud M Lifante J C Bilateral adrenalectomy in Cushing's disease: Altered long-term quality of life compared to other treatment options Annales d'endocrinologie 2019 80 32 37 10.1016/j.ando.2018.01.002 30243473

[33] Bauer J Klingelhöfer D Maier W Spatial accessibility of general inpatient care in Germany: an analysis of surgery, internal medicine and neurology Scientific reports 2020 10 19157 10.1038/s41598-020-76212-0 PMC7645718 33154470

[34] Launay L Guillot F Gaillard D Methodology for building a geographical accessibility health index throughout metropolitan France PLoS One 2019 14 e0221417 10.1371/journal.pone.0221417 PMC6705764 31437261

[35] Deutsche Diabetes Gesellschaft e.V. (DDG) Deutscher Gesundheitsbericht Diabetes 2022

[36] Jaursch-Hancke C Stationäre Versorgung von Menschen mit Diabetes und endokrinologischen Erkrankungen zunehmend in Gefahr Der Diabetologe 2020 7 685

[37] Deutsche Diabetes Gesellschaft e.V. (DDG) und Deutsche Gesellschaft für Endokrinologie (DGE). Endokrinologie und Diabetologie: Versorgungsengpässe sind vorprogrammiert, jetzt gegensteuern!2022

[38] Johanssen S Hahner S Saeger W Deficits in the management of patients with adrenocortical carcinoma in Germany Dtsch Arztebl Int 2010 107 885 891 10.3238/arztebl.2010.0885 PMC3021904 21246024

[39] Moebus S Hanisch J Bramlage P Regional differences in the prevalence of the metabolic syndrome in primary care practices in Germany Dtsch Arztebl Int 2008 105 207 13 10.3238/artzebl.2008.0207 PMC2696781 19629210

[40] Ahlqvist E Storm P Karajamaki A Novel subgroups of adult-onset diabetes and their association with outcomes: a data-driven cluster analysis of six variables The lancet Diabetes & endocrinology 2018 6 361 369 10.1016/S2213-8587(18)30051-2 29503172

[41] Hedley V Bottarelli V Weinman A Shaping national plans and strategies for rare diseases in Europe: past, present, and future Journal of Community Genetics 2021 12 207 216 10.1007/s12687-021-00525-4 PMC8097119 33950416

[42] Guimarães J Afonso A Carvalho D [Endocrinology in Portugal - Census 2016. Board of the Portuguese College of Endocrinology and Nutrition of the Portuguese Medical Association] Acta medica portuguesa 2017 30 642 651 10.20344/amp.8379 29025531

